# A Rare Case of Metastatic Choriocarcinoma of Lung Origin

**DOI:** 10.1155/2017/4649813

**Published:** 2017-12-07

**Authors:** Parth Rali, Jianwu Xie, Grishma Rali, Mayur Rali, Jan Silverman, Khalid Malik

**Affiliations:** ^1^Division of Pulmonary and Critical Care, Allegheny General Hospital, Pittsburgh, PA 15212, USA; ^2^Division of Pathology, Allegheny General Hospital, Pittsburgh, PA 15212, USA; ^3^Children's Hospital of Philadelphia, Philadelphia, PA, USA; ^4^Hofstra Northwell School of Medicine, Department of Family Medicine, Southside Hospital, Bay Shore, NY, USA

## Abstract

Choriocarcinoma is part of the spectrum of gestational trophoblastic disease that occurs in women of reproductive age. Although the most common metastatic site of choriocarcinoma is the lung, primary pulmonary choriocarcinoma is rare. To diagnose primary pulmonary choriocarcinoma, the patient should have no previous gynecologic malignancy, have elevated human chorionic gonadotropin, and have pathological confirmation of the disease excluding gonadal primary site of the tumor. Due to the paucity of data, there are no guidelines for treatment. Prognosis of this malignancy is extremely poor. We report a rare case of metastatic primary lung choriocarcinoma in a 69-year-old postmenopausal woman who was treated with combination of surgery, chemotherapy, and radiation. The patient had a good outcome and is doing well after 1-year follow-up.

## 1. Introduction

Choriocarcinoma is a germ cell tumor containing cells of trophoblastic origin. It is usually associated with gestational event like molar pregnancy and secretes human chorionic gonadotropin (b-hCG) [1]. Primary pulmonary choriocarcinoma (PPC) is a rare tumor that generally affects young individuals [2]. It is rarely reported in postmenopausal women [3]. PPC portends a very poor prognosis with a dismal 5-year survival rate of <5% [3]. We report a rare case of metastatic PPC in a 69-year-old postmenopausal woman.

## 2. Case

**Figure 1:**
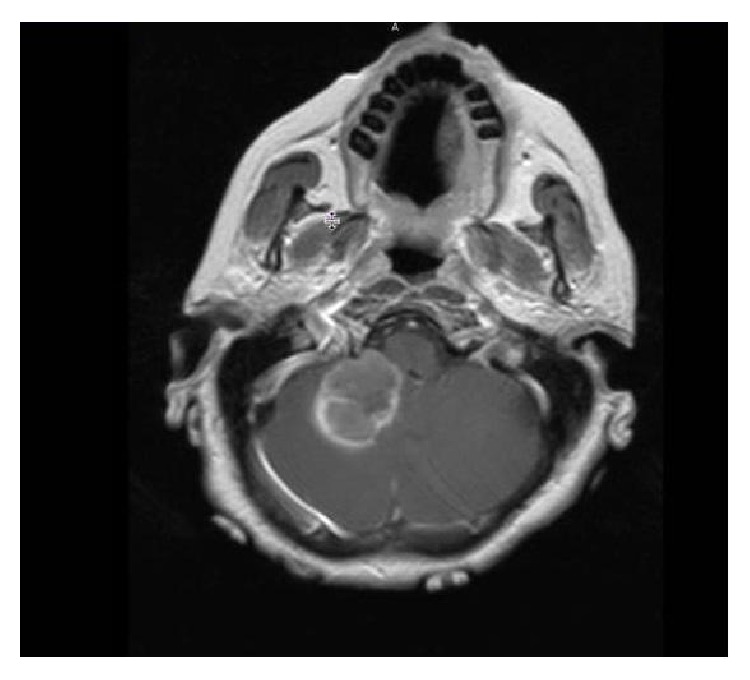
T1 weighted MRI of brain showing right cerebellar lesion.

69-year-old-woman with history of hypertension, hyperlipidemia, and 50 pack-year smoking presented to our emergency room with 8 days of new onset dizziness, gait instability, and severe occipital headaches. Physical examination was positive for horizontal nystagmus to the right. CT scan of her head demonstrated a right sided inferior cerebellar lesion with minimal hemorrhage which was confirmed on the MRI of the brain (Figure 1). Patient was started on decadron and admitted to neurosurgical intensive care unit. Patient underwent skull base suboccipital craniectomy with microscopic resection and debulking of the cerebellar mass. Follow-up CT head showed no residual tumor and patient had a complete clinical recovery.

**Figure 2:**
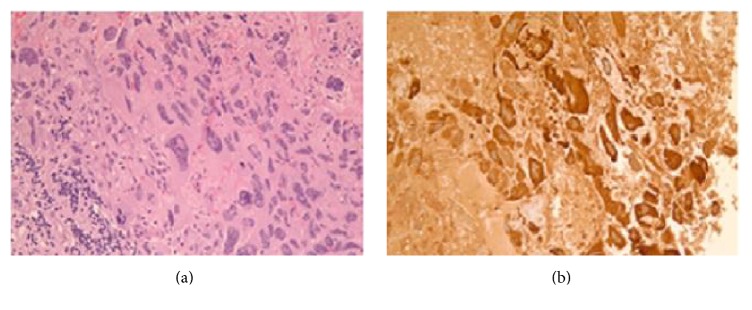
Brain biopsy. (a) Histologic examination of the biopsy reveals a sheet of syncytiotrophoblastic cells characterized by multilobulated to multinucleated and spindle shaped cells with a moderate amount of eosinophilic cytoplasm (H&E ×20). (b) Immunohistochemical stain for beta-hCG shows moderate to strong cytoplasmic staining of the syncytiotrophoblastic cells (×20).

**Figure 3:**
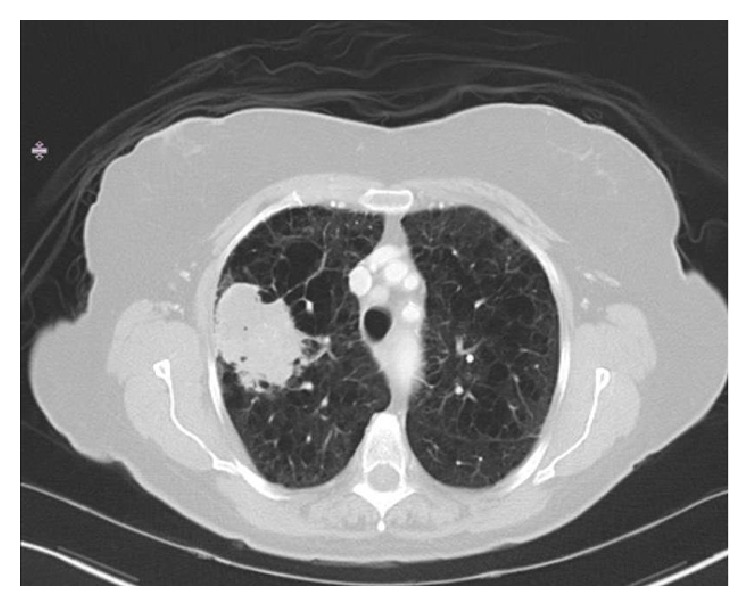
CT chest showing right upper lobe mass with emphysema.

Histologic examination of the brain metastasis was consistent with syncytiotrophoblastic cells. Immunohistochemical stains were positive for cytokeratin 5.2, AE1/AE3, EMA, and CK7 and strongly positive for beta-hCG supportive for choriocarcinoma differentiation (Figure 2). Metastatic workup which included CT chest, abdomen, and pelvis and PET scan showed a right upper lobe (RUL) mass (Figure 3). There was no evidence of any other metastatic disease. The serum b-hCG was elevated to 1668 mIU/ml (normal <10 mIU/ml).

**Figure 4:**
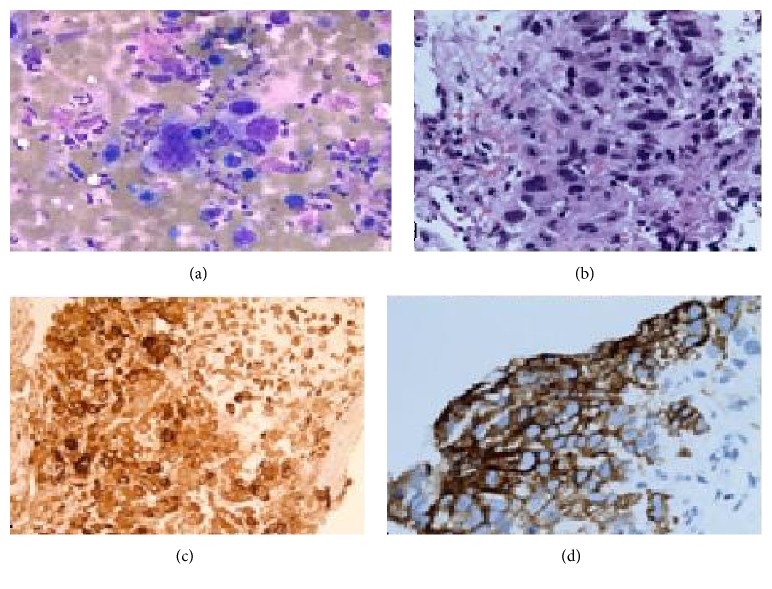
RUL mass biopsy. (a) Touch imprint cytology from core needle biopsy demonstrates bizarre, pleomorphic cells arranged in a dissociative fashion. Some of the tumor cells are multilobulated to multinucleated with pleomorphic, hyperchromatic nuclei (Diff-Quik stain ×20). (b) Core needle biopsy reveals bizarre syncytiotrophoblasts characterized by pleomorphic cells, including some that are multilobulated to multinucleated. The cells have a moderate amount of surrounding eosinophilic cytoplasm. Extensive necrosis is noted in the cores (H&E ×20). (c) Immunohistochemical stain for beta-hCG shows intense strong positive cytoplasmic staining (×40). (d) Strong diffuse positive cytoplasmic and membranous staining for PLAP (×40).

CT guided core needle biopsy of the RUL lung mass and touch imprint cytology demonstrated poorly differentiated malignant cells, including scattered multinucleated tumor giant cells. Immunohistochemical studies performed on the core biopsy demonstrated positive staining of the malignant cells for PLAP and beta-hCG. The malignant cells were negative for AFP, CD30, OCT 3/4, glypican-3, and CD-117 which supports the diagnosis of a poorly differentiated lung carcinoma with choriocarcinomatous differentiation. The negative staining for TTF-1, Napsin-A, CK 5/6, and p63 supports that the poorly differentiated malignancy is neither a conventional adenocarcinoma nor squamous cell carcinoma, respectively (Figure 4).

The patient had a follow-up normal pelvic examination, endometrial biopsy, and pelvic ultrasound. The patient refused to undergo lung resection of her tumor. She was treated with whole brain and lung mass radiation and 6 cycles of chemotherapy with carboplatin and etoposide. With chemoradiation, her b-hCG level continued to decrease. She had persistence of the right upper lobe mass without any evidence of metastatic disease on subsequent follow-up imaging. Long-term plans are close clinical follow-up with serial b-hCG and imaging.

## 3. Discussion

Choriocarcinoma is a malignant proliferation of trophoblastic cells. The histological pattern is characterized by sheets of cytotrophoblasts and syncytiotrophoblast without chorionic villi [4]. Choriocarcinoma is part of the spectrum of gestational trophoblastic disease and it commonly occurs in women of reproductive age [4, 5].

Trophoblastic diseases have affinity for blood vessels and usually metastasize hematogenously [5]. The most common metastatic site is the lung (80%) and the occurrence of respiratory failure requiring intubation is an independent factor for poor outcome. Metastasis to the brain occurs in 10% of patients and virtually all patients with cerebral involvement have a simultaneous pulmonary involvement [4].

Primary lung carcinoma with trophoblastic differentiation is rare. Although cases of PPC have been reported as early as 1950s [6, 7], there are at least 34 cases reported including this case [8], although the exact number of cases is uncertain since similar cases may have been reported with different names.

The diagnostic criteria proposed include [1] no previous gynecologic malignancy, solitary or predominant lung lesion with the exclusion of a gonadal primary site, elevated b-hCG titers that normalize after surgery or chemotherapy, and pathologic confirmation of the disease

The major differential diagnosis is giant cell carcinoma of lung, since both malignancies are composed of a dimorphic population of polygonal mononucleated and pleomorphic, multinucleated giant cells. Some authors considered that the presence of predominant tumor multinucleated giant cells in tumor tissue as a diagnostic feature for a primary lung choriocarcinoma and sheets of polygonal mononucleated tumor cells with scattered giant cells as a feature of giant cell carcinoma of lung [9]. By immunohistochemical stain, the giant cells in primary lung choriocarcinoma show very strong positive staining of b-HCG, as seen in our case [9]. However, the possibility of a mix germ cell tumor cannot be totally excluded based on the lung biopsy, but no other germ cell components were seen in the resected brain metastasis.

The understanding of the cell origin and pathogenesis of primary lung choriocarcinoma is limited, though multiple theories exist [9]. The first popular theory is that it arises from an ectopic primordial germ cell in the lung during the embryonic development. The second theory is that the primary lung choriocarcinoma is a high-grade transformation from a nontrophoblastic lung tumor. The third theory is that the primary lung choriocarcinoma and giant cell carcinoma of lung are the same entity. Although all these theories are reasonable, molecular studies might prove useful to better define the histogenesis of this rare malignancy.

In our case, a negative CT scan of the abdomen and pelvis, cervical cytology, and endometrial biopsy ruled out gynecologic origin of the tumor. The patient's age and clinical presentation also make it unlikely to be from the gynecological tract. Serum b-hCG titer decreased to value of 98 mIU/mL after a one-year follow-up.

Due to paucity of data, there is no guideline for treatment of PPC. Different treatment modalities have been used with varying success. Surgery alone or with chemo and radiation therapy seems to have a better outcome [9, 10].

In our patient, chemotherapy regimen included etoposide and carboplatin. Etoposide served as the primary agent to induce remission in chorionic tumors [4]. Chemotherapy regimen with etoposide and platinum has been associated with good result. The patients were alive on 1-year follow-up just like our patient [1, 2]; however, the prognosis of PPC remains very poor with a <5% of patients surviving after 5 years [3].

## References

[1] Di Crescenzo V., Laperuta P., Napolitano F., Carlomagno C., Garzi A., Vitale M. (2013). An unusual case of primary choriocarcinoma of the lung. *BMC Surgery*.

[2] Kinni U. B. M. (2007). Primary pulmonary choriocarcinoma: Is it still an enigma. *Indian Journal of Chest Diseases and Allied Sciences*.

[3] Serno J., Zeppernick F., Jäkel J. (2012). Primary pulmonary choriocarcinoma: Case report and review of the literature. *Gynecologic and Obstetric Investigation*.

[4] Berkowitz R. S., Goldstein D. P. (1996). Chorionic tumors. *The New England Journal of Medicine*.

[5] Li X. M., Liu X. Y., Liu Z. X. (2015). Choriocarcinoma with multiple lung, skull and skin metastases in a postmenopausal female: A case report. *Oncology Letters*.

[6] Brouet G., Chretien J., Marche J., Roussel G. (1959). Apparently primary pulmonary choriocarcinoma in pregnancy. *Journal français de médecine et chirurgie thoraciques*.

[7] Schulz M., Hernandez A., Rebora F. (1956). Primary chorio-carcinoma of the lung; Case report. *Rev Mex Tuberc Enferm Apar Respir*.

[8] Ikura Y., Inoue T., Tsukuda H., Yamamoto T., Ueda M., Kobayashi Y. (2000). Primary choriocarcinoma and human chorionic gonadotrophin-producing giant cell carcinoma of the lung: Are they independent entities?. *Histopathology*.

[9] Kamata S., Sakurada A., Sato N., Noda M., Okada Y. (2016). A case of primary pulmonary choriocarcinoma successfully treated by surgery. *General Thoracic and Cardiovascular Surgery*.

[10] Vaideeswar P., Mehta J., Deshpande J. (2004). Primary pulmonary choriocarcinoma - A series of 7 cases. *Indian Journal of Pathology and Microbiology*.

